# Supplementary material for: Competitive intelligence and its impact on innovations in tourism industry of China: An empirical research
Source: PLoS One. 2020 Jul 31;15(7):e0236412. doi: 10.1371/journal.pone.0236412 (PMC7394418; doi:10.1371/journal.pone.0236412)
Supplement: S1 Data — (DOCX) [file pone.0236412.s001.docx]

**调查问卷**

尊敬的先生/女士:

您好!非常感谢您在百忙之中。抽出时间参与我们的调查。本问卷旨在研究我国旅游企业进行服务创新与创新绩效之间的作用关系，**答案没有对错之分。**请选择与您想法/感受最为接近的答案。

本问卷纯属学术研究之用，所获信息也绝不用于任何商业目的，请您放心并尽可能的根据实际情况客观回答。如果您对本问卷的结论感兴趣，请在问卷结尾处标注并留下您的联系方式，我们会将研究成果及时发给您。

以下是对贵企业近三年经营管理情况的描述： 1=非常不同意，2=不同意，3=比较不同意，4=不好确定，5=比较同意，6=同意，7=非常同意。

| 序号 | 题项 | 1 | 2 | 3 | 4 | 5 | 6 | 7 |
| --- | --- | --- | --- | --- | --- | --- | --- | --- |
| Customer CI refers to the competitive intelligence about customers: 顾客情报是指与顾客有关的竞争情报。 | | | | | | | | |
| 1 | CCI1 We collect basic information of customers, including their name, age, occupation/profession,et.al. 我们收集客户的基本信息，包括他们的姓名、年龄、职业等。 |  |  |  |  |  |  |  |
| 2 | CCI2 We collect and analyze customer need/demand about our services.我们收集并分析客户对我们服务的需求。 |  |  |  |  |  |  |  |
| 3 | CCI3 We collect and analyze customer satisfaction and customer complaints.我们收集和分析客户满意度和客户投诉情况。 |  |  |  |  |  |  |  |
| 4  4 | CCI4 We invite customers to participate in service innovation.我们邀请客户参与服务创新。 |  |  |  |  |  |  |  |
| Opponent CI refers to the competitive intelligence about opponents:竞争对手情报是指关于竞争对手的情报： | | | | | | | | |
| 5 | OCI1 We collect and analyze opponents’ daily operation information. 我们收集并分析对手的日常运营信息。 |  |  |  |  |  |  |  |
| 6 | OCI2 We keep eyes on R&D progress of our opponents.我们关注对手的研发进展。 |  |  |  |  |  |  |  |
| 7 | OCI3 We pay attention to marketing of the new services/products from our opponents. 我们关注对手推销新服务/产品的情报。 |  |  |  |  |  |  |  |
| Supplier CI refers to the competitive intelligence about suppliers:供应商情报是指关于供应商的竞争情报： | | | | | | | | |
| 8 | SCI1 We collect and analyze information about inventory of suppliers’ service/products.我们收集和分析有关供应商服务/产品库存的信息。 |  |  |  |  |  |  |  |
| 9 | SCI2 We collect and analyze information about R&D of new service/products of suppliers.我们收集和分析供应商新服务/产品的研发信息。 |  |  |  |  |  |  |  |
| 10 | SCI3 We collect and analyze information about marketing of service/products supplied by our suppliers. 我们收集并分析供应商提供的服务/产品的市场信息。 |  |  |  |  |  |  |  |
| Exploitative service innovation(ETSI) refers to a small-scale and gradual innovation activity with the intention to improve the existing status. 利用式服务创新是一种小幅度、渐进的创新活动，其意图是对服务现状进行改进。 | | | | | | | | |
| 11 | ETSI1 We make efforts to improve the applicability of existing service/skills in many related business areas.我们努力提高已有的技术/技能在多个相关业务领域的适用性。 |  |  |  |  |  |  |  |
| 12 | ETSI2 We often use existing service/skills to increase the functions and types of products/services. 我们经常利用已有的技术/技能来增加产品/服务的功能和种类。 |  |  |  |  |  |  |  |
| 13 | ETSI3 We often improve existing service/skills to meet current needs.我们经常对已有的技术/技能进行改良，以适应当前需要。 |  |  |  |  |  |  |  |
| 14 | ETSI4 We often refine our accumulated business experience and applies it to the current business. 我们经常对公司积累的业务经验进行提炼，并应用于当前业务中。 |  |  |  |  |  |  |  |
| Exploratory Service innovation(ERSI) refer to a large-scale and radical innovation activity with the intention of finding new possibilities. 探索式创新是一种大幅度的、激进的创新活动，其意图是寻找新的可能性。 | | | | | | | | |
| 15 | ERSI1 We frequently develop brand-new market segments without relevant marketing experience. 我们经常开拓全新的、尚无相关营销经验的细分市场。 |  |  |  |  |  |  |  |
| 16 | ERSI2 We often adopt business strategies/tactics that have not been adopted by other companies in the same industry. 我们经常采用同行业其他公司没有采用过的经营战略/战术。 |  |  |  |  |  |  |  |
| 17 | ERSI3 We frequently use immature and risky new services/skills. 我们经常运用尚不成熟、有一定风险的新技术/技能。 |  |  |  |  |  |  |  |
| 18 | ERSI4 We frequently develop new and radical products/services. 我们经常开发全新的、根本性变革的产品/服务。 |  |  |  |  |  |  |  |
