# Supplementary material for: Competitive intelligence and its impact on innovations in tourism industry of China: An empirical research
Source: PLoS One. 2020 Jul 31;15(7):e0236412. doi: 10.1371/journal.pone.0236412 (PMC7394418; doi:10.1371/journal.pone.0236412)
Supplement: S2 Data — (DOCX) [file pone.0236412.s002.docx]

**Ethics Statement**

The study was approved and supported by the institutional review board of Wuxi Tourism Association, Jiangsu, China. The subject of this manuscript is CI and service innovation situation in companies rather than human beings. All people interviewed with the questionnaire provided their consent by answering the questions. Their names and personal information are kept secret. Therefore, they are free to express their feelings about the CI and service innovation in their companies.
